# Corneal higher-order aberrations of the anterior surface, posterior surface, and total cornea after small incision lenticule extraction (SMILE): high myopia versus mild to moderate myopia

**DOI:** 10.1186/s12886-018-0965-1

**Published:** 2018-11-12

**Authors:** Hong-Ying Jin, Ting Wan, Xiao-Ning Yu, Fang Wu, Ke Yao

**Affiliations:** Eye Center, 2nd Affiliated Hospital, School of Medicine, Zhejiang University, Hangzhou, 310009 China

**Keywords:** SMILE, Higher-order aberrations, Posterior cornea

## Abstract

**Background:**

To investigate corneal higher-order aberrations (HOAs) of the anterior surface, posterior surface, and total cornea after small incision lenticule extraction (SMILE) in high myopic and mild to moderate myopic patients.

**Methods:**

This retrospective study included 197 eyes (101 patients) undergoing SMILE surgery. According to the preoperative spherical equivalent (SE), treated eyes were divided into two groups: a high myopic group (more than − 6.0 D, Group H) and a mild to moderate myopic group (less than − 6.0 D, Group M). Corneal HOAs of the anterior surface, posterior surface, and total cornea were measured using a Scheimpflug camera preoperatively and 3 months postoperatively. Pearson’s correlation analysis was conducted to determine relationships between corneal aberrations and the SE.

**Results:**

There were no significant differences in third-order to eight-order aberrations (RMS HOAs) of the anterior surface, posterior surface, and total corneal between the two groups before SMILE surgery. However, after SMILE, anterior and total corneal HOAs, especially vertical coma and spherical aberrations, significantly increased in both groups (*p* < 0.0167), whereas posterior corneal HOAs remained relatively stable (*p* > 0.0167). The induction of HOAs was significantly greater in Group H than Group M postoperatively (*p* < 0.0167). Changes in anterior surface and total corneal HOAs, especially vertical coma and spherical aberrations, were related to the SE (*p* < 0.05).

**Conclusions:**

Anterior and total corneal HOAs, particularly vertical coma and spherical aberrations, significantly increased after SMILE in both groups, whereas posterior corneal HOAs remained stable. Aberration changes were related to SE.

**Trial registration:**

Retrospectively registered. ChiCTR-ORC-17011040. Registered 1 April 2017. Name of registry: The observation of clinical results after corneal refractive surgery. Data of enrolment of the first participant to the trial: 15 December 2016.

## Background

Visual acuity is the most commonly used parameter for assessments of overall visual function in the clinical setting. However, under dim light conditions or low contrast sensitivity conditions, visual acuity does not truly reflect a patient’s subjective vision function [1], especially after corneal refractive surgeries. Higher-order aberrations (HOAs) increase after successful refractive surgery and influence the vision quality of patients. Measurements of changes in wavefront aberrations are used to evaluate the impact of refractive surgery on vision quality [1]. Previous studies indicated that posterior corneal HOAs influenced corneal biomechanical responses and provided valuable information in determining the cause of poor vision quality after corneal refractive surgery [2, 3].

Small incision lenticule extraction (SMILE) surgery is a newly developed corneal refractive technique, which is less invasive than LASIK (Laser-assisted in situ keratomileusis) and FS-LASIK (femtosecond laser-assisted LASIK), with only a small incision required [4, 5]. Recent studies suggested that the SMILE procedure provided excellent clinical outcomes, considering its safety, efficacy, predictability, and postoperative ocular surface health [6–8]. Therefore, SMILE is considered to be a good choice for refractive surgery. Previous studies of changes of corneal HOAs after the SMILE procedure focused mainly on anterior corneal HOAs or total corneal HOAs [8–11]. Only a few studies have compared anterior, posterior, and total corneal HOAs following different refractive surgery procedures [2, 3, 12]. The potential effect of the SMILE procedure on posterior corneal HOAs is not well understood. In this study, we compared HOAs of the anterior surface, posterior surface, and total cornea after SMILE surgery for high myopia and mild to moderate myopia.

## Material and methods

### Subjects

This is a retrospective study, which recruited 197 eyes (101 patients). All patients aged between 18 and 47 years underwent SMILE surgery at the Department of Ophthalmology, Second Affiliated Hospital, College of Medicine, Zhejiang University from December 2016 to May 2017 were included in this study. This research followed the tenets of the Declaration of Helsinki was approved by the Institutional review board (No: 2017–017). Informed written consent was obtained from the all subjects. The inclusion criteria were aged ≥18 years, myopic spherical correction < − 10.00 diopters, no ocular or systemic diseases, and stable refraction for at least one year. The exclusion criteria were the presence of active ocular disease or a history of ocular surgery and trauma. Patients were instructed to stop wearing soft contact lenses for at least 1 week prior to the surgery. The cases were divided into two groups according to the degree of preoperative spherical equivalent (SE): a high myopic group (more than − 6.0 D,Group H) and a mild to moderate myopic group (less than − 6.0 D, Group M).

### Surgical technique

All surgeries were performed by the same experienced surgeon (H.Y.J.). A VisuMax femtosecond laser system (Carl Zeiss Meditec AG, Jena, Germany) was used for surgical refractive corrections in all patients, with a repetition rate of 500 kHz and pulse energy of 155 nJ. The stroma cap was set at thickness of 120 μm, diameter of 7.5–7.6 mm. Prior to the initiation of suction, the patients were instructed to fixate on a target light. Four cleavage planes were created, including anterior and posterior surfaces of the refractive lenticule and vertical edge of the refractive lenticule. A single side-cut incision (width of 2 mm) was made at an angle of 120°. The lenticule was removed using a forceps. The target refraction was emmetropia. After surgery, all the patients received a topical antibiotic for 7 days and a topical steroid for 2 weeks. Artificial tears were used for more than 4 weeks.

### Measurement of corneal aberrations

Corneal aberrations of the anterior surface, posterior surface, and total cornea were measured by a rotating Scheimpflug Camera (Pentacam HR; Oculus, Wetzlar, Germany). The Pentacam HR is a noninvasive and reproducible diagnostic method. The measurements were made in a dark room. To minimize the potential effect of tear film on corneal imaging, the patients were instructed to remain fixated on a target light immediately after blinking. The Pentacam HR camera then started rotating and scanning the cornea. Only measurements marked as “OK” quality were considered valid. Corneal aberrations of the anterior surface, posterior surface, and total cornea were analyzed over a 6.0-mm central diameter preoperatively, 1 and 3 months postoperatively. Root mean square (RMS) values of higher-order aberrations (HOAs) (third-order to eight-order), including coma, trefoil, quadrafoil, and secondary astigmatism, were calculated.

### Statistical analysis

Statistical analyses were performed using SPSS software, ver. 18 (SPSS, Chicago, IL, USA). The Kolmogorov–Smirnov test was used to test for normality. An independent-sample *t* test was conducted for comparisons between Group H and Group M. A paired-sample *t* test was performed for preoperative and postoperative comparisons. The Bonferroni correction for multiple testing was used to reduce the rate of type I error. In addition, Pearson’s correlation analysis was performed to determine relationships between the preoperative spherical equivalent (SE) and corneal aberrations. All values are given as the mean ± standard deviation. *P* < 0.05 was considered statistically significant, otherwise indicated.

## Results

All 101 patients attended the 1-month, and 3-month follow-up examinations. Group H and Group M comprised 65 and 132 eyes, respectively. Preoperative characteristics of both groups are listed in Table 1. There were no statistically significant differences between the two groups as regards the patient’s age, preoperative SE, spherical diopter, cylindrical diopter, central corneal thickness(CCT), intraocular pressure(IOP), and mean corneal power, making it possible to compare corneal HOAs without confounders (*p* > 0.05).

**Table 1:** Demographic and preoperative patient information (mean ± SD)

Parameter	Group H	Group M	t	*p*
Eye (*n*)	65	132		
Sex (M/F)	31/34	77/56		
Age (y)	24.46 ± 7.34 (18 to 47)	23.84 ± 5.92 (18 to 47)	0.64	0.52
IOP (mmHg)	15.80 ± 2.12 (11 to 23)	15.48 ± 2.36 (10 to 23)	0.93	0.36
CCT (μm)	552.58 ± 25.61 (502 to 616)	541.52 ± 28.03 (482 to 627)	1.66	0.10
Mean corneal power (D)	43.16 ± 1.42 (40.60 to 46.55)	43.24 ± 1.25 (41.25 to 45.95)	−0.42	0.67
SE (D)	−7.32 ± 0.99 (−6.00 to −9.88)	−4.45 ± 1.01 (− 1.13 to − 5.88)	− 18.85	0.00*
Sphere (D)	−6.94 ± 1.00 (− 5.00 to − 9.75)	−4.13 ± 1.00 (− 0.75 to − 5.75)	− 18.60	0.00
Cylinder (D)	−0.76 ± 0.68 (0 to − 3.00)	−0.64 ± 0.50 (0 to − 2.75)	−1.35	0.18
Lenticule thickness (μm)	128.48 ± 10.27 (109 to148)	92.23 ± 15.53 (50 to 120)	19.51	0.00*
Lenticule diameter (mm)	6.52 ± 0.14 (6.1 to 6.6)	6.58 ± 0.05 (6.1 to 6.6)	−5.84	0.00*

*SD* standard deviation, *D* diopters, *SE* spherical equivalent, *CCT* central corneal thickness, *IOP* intraocular pressure, Group *H* high myopia group, Group *M* mild to moderate myopia group.*p < 0.05

### Comparison of surgically induced third-order to eight-order aberrations (RMS HOAs) after SMILE in group H and group M

**Fig. 1:**
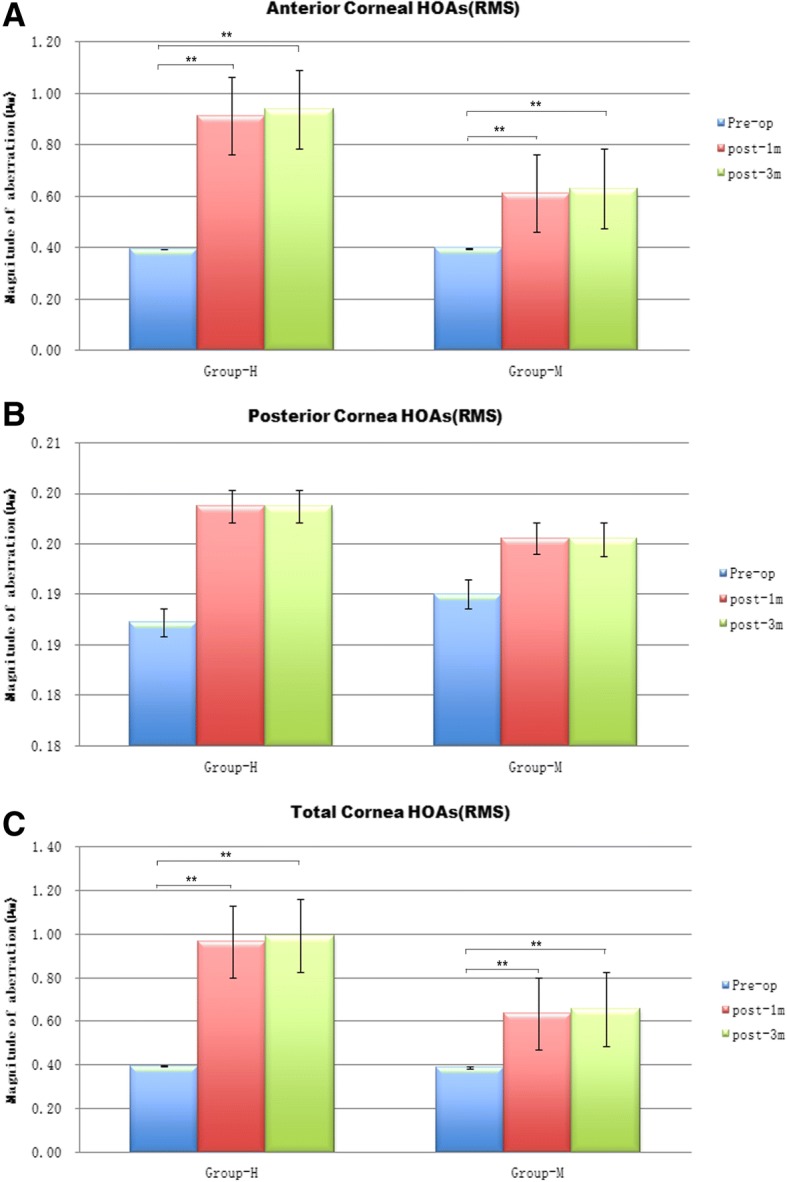
Third-order to eight-order higher-order aberrations (RMS HOAs) of the cornea preoperatively and 1 and 3 months postoperatively in Group H and Group M. **a** Anterior corneal surface (RMS-HOA-CF, root mean square –HOA- cornea front); **b** Posterior corneal surface (RMS-HOA-CB, root mean square-HOA- cornea back); **c** Total cornea (RMS-HOA-cornea, root mean square- HOA- cornea). *p < 0.0167 (the modified Bonferroni correction, 0.05/3 tests)

As shown by RMS values, third-order to eight-order aberrations of the anterior corneal surface (RMS-HOA-CF) and total cornea (RMS-HOA-cornea) significantly increased after SMILE surgery in Group H and Group M (*p* < 0.0167) (Fig. 1a and c). According to RMS values, posterior corneal HOAs (RMS-HOA-CB) slightly increased, but remained relatively stable in both groups (*p* > 0.0167) (Fig. 1b).

### Comparison of preoperative HOAs versus surgically induced HOAs (third-order to eight-order, RMS HOAs) between group H and group M

As shown by RMS values, there were no significant differences in HOAs between Group H and Group M preoperatively (*p* > 0.0167). However, there were significant differences in HOAs in anterior cornea and total cornea between the two groups 1 and 3 months after surgery (*p* < 0.0167). There was no significant difference in HOAs of posterior cornea surface between the two groups after the surgery (*p* > 0.0167) (Table 2).

**Table 2:** Comparison of higher-order aberrations (HOAs) between the two groups preoperatively and postoperatively 1 and 3 months (mean ± SD)

Time	Preop.			Postop. 1 month			Postop. 3 months		
Group	Group H (*n* = 65)	Group M (*n* = 132)	*p*	Group H (*n* = 65)	Group M (*n* = 132)	*p*	Group H (*n* = 65)	Group M (*n* = 132)	*P*
RMS HOA (CF)	0.39 ± 0.08	0.40 ± 0.11	0.754	0.91 ± 0.28	0.61 ± 0.18	0.000*	0.94 ± 0.26	0.63 ± 0.19	0.000^#^
RMS HOA (CB)	0.19 ± 0.03	0.19 ± 0.03	0.546	0.20 ± 0.03	0.20 ± 0.03	0.219	0.19 ± 0.03	0.20 ± 0.03	0.640
RMS HOA (cornea)	0.40 ± 0.09	0.39 ± 0.12	0.710	0.97 ± 0.30	0.64 ± 0.19	0.000	0.99 ± 0.28	0.66 ± 0.20	0.000^#^
Oblique trefoil Z^3,3^ (CF)	0.00 ± 0.07	− 0.01 ± 0.09	0.294	0.01 ± 0.10	− 0.01 ± 0.09	0.486	0.03 ± 0.11	0.01 ± 0.11	0.376
Horizontal coma Z^3,1^ (CF)	−0.01 ± 0.15	− 0.03 ± 0.15	0.304	0.01 ± 0.37	− 0.04 ± 0.25	0.434	− 0.03 ± 0.38	− 0.05 ± 0.26	0.996
Vertical coma Z ^3,-1^ (CF)	−0.03 ± 0.19	0.01 ± 0.18	0.276	−0.60 ± 0.31	− 0.34 ± 0.23	0.000*	− 0.62 ± 0.29	−0.32 ± 0.26	0.000^#^
Vertical trefoil Z^3,-3^ (CF)	−0.04 ± 0.10	−0.05 ± 0.11	0.666	−0.03 ± 0.13	− 0.02 ± 0.11	0.988	− 0.04 ± 0.12	− 0.03 ± 0.13	0.661
Spherical aberration Z^4,0^ (CF)	0.24 ± 0.07	0.26 ± 0.07	0.188	0.44 ± 0.14	0.31 ± 0.11	0.000*	0.45 ± 0.13	0.30 ± 0.11	0.000#
Oblique trefoil Z^3,3^ (CB)	− 0.01 ± 0.05	0.00 ± 0.04	0.619	0.00 ± 0.05	− 0.01 ± 0.04	0.490	− 0.01 ± 0.04	−0.01 ± 0.05	0.895
Horizontal coma Z^3,1^ (CB)	0.00 ± 0.02	0.00 ± 0.02	0.408	− 0.01 ± 0.03	0.00 ± 0.03	0.180	−0.01 ± 0.03	0.00 ± 0.03	0.461
Vertical coma Z^3,-1^ (CB)	−0.02 ± 0.03	−0.03 ± 0.04	0.025	0.01 ± 0.04	− 0.01 ± 0.04	0.000*	0.01 ± 0.03	−0.01 ± 0.04	0.000^#^
Vertical trefoil Z^3,-3^ (CB)	−0.02 ± 0.05	−0.02 ± 0.05	0.220	−0.05 ± 0.05	− 0.04 ± 0.06	0.249	− 0.04 ± 0.05	−0.04 ± 0.05	0.470
Spherical aberration Z^4,0^ (CB)	−0.15 ± 0.03	−0.16 ± 0.03	0.306	−0.16 ± 0.03	− 0.16 ± 0.03	0.821	− 0.16 ± 0.03	−0.16 ± 0.03	0.857
Oblique trefoil Z^3,3^ (cornea)	0.00 ± 0.09	− 0.02 ± 0.10	0.506	0.01 ± 0.11	−0.01 ± 0.09	0.250	0.02 ± 0.12	0.00 ± 0.12	0.339
Horizontal coma Z^3,1^ (cornea)	−0.01 ± 0.15	−0.03 ± 0.15	0.307	0.00 ± 0.39	− 0.05 ± 0.26	0.457	− 0.04 ± 0.40	−0.06 ± 0.27	0.964
Vertical coma Z^3,-1^ (Cornea)	−0.04 ± 0.20	−0.01 ± 0.18	0.351	−0.66 ± 0.23	− 0.39 ± 0.23	0.000*	− 0.68 ± 0.31	−0.36 ± 0.27	0.000^#^
Vertical trefoil Z^3,-3^ (cornea)	−0.07 ± 0.12	−0.07 ± 0.12	0.864	−0.08 ± 0.14	− 0.06 ± 0.12	0.600	− 0.08 ± 0.14	−0.07 ± 0.14	0.454
Spherical aberration Z^4,0^ (cornea)	0.19 ± 0.08	0.20 ± 0.07	0.398	0.41 ± 0.16	0.26 ± 0.12	0.000*	0.42 ± 0.14	0.26 ± 0.13	0.000^#^

*Significant difference in HOAs 1 months postoperatively (*p* < 0.0167) *(the modified Bonferroni correction, 0.05/3 tests)* between Group-H and Group-M; ^#^Significant difference in HOAs 3 months postoperatively (*p* < 0.0167) *(the modified Bonferroni correction, 0.05/3 tests)* between Group-H and Group-M. *HOAs* higher-order aberrations, *RMS* root mean square, RMS-HOA-CF: third-order to eight-order aberrations of anterior corneal surface (cornea front, CF); RMS-HOA-cornea: third-order to eight-order aberrations of total corneal surface; RMS-HOA-CB: third-order to eight-order aberrations of posterior corneal surface (cornea back, CB)

### Comparison of surgically induced anterior corneal surface HOAs in group H and group M

**Fig. 2:**
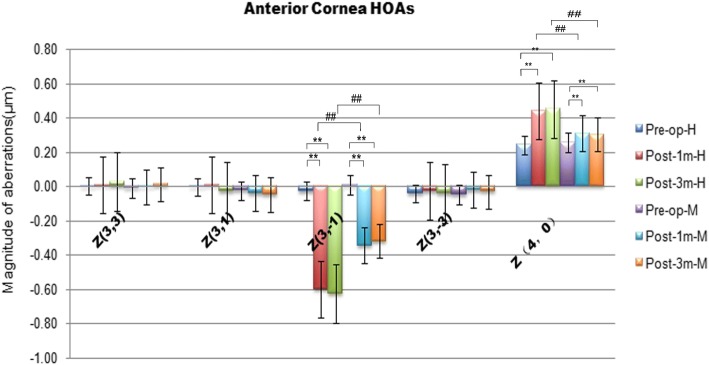
Higher-order aberrations (HOAs) of the anterior cornea surface before and after SMILE surgery in Group H and group M. *p, ^#^*p* < 0.0167 (the modified Bonferroni correction, 0.05/3 tests)

There were no significant differences in coma and spherical aberrations of the anterior surface between the two groups preoperatively (*p* > 0.0167). However, there was a significant increase in vertical coma (Z^3,-1^) and spherical aberrations (Z^4, 0^) in the two groups 1 and 3 months postoperatively (*p* < 0.0167). More coma and spherical aberrations of the anterior corneal surface were induced in Group H than Group M postoperatively (*p* < 0.0167) (Fig. 2).

### Comparison of surgically induced posterior corneal surface HOAs in group H and group M

**Fig. 3:**
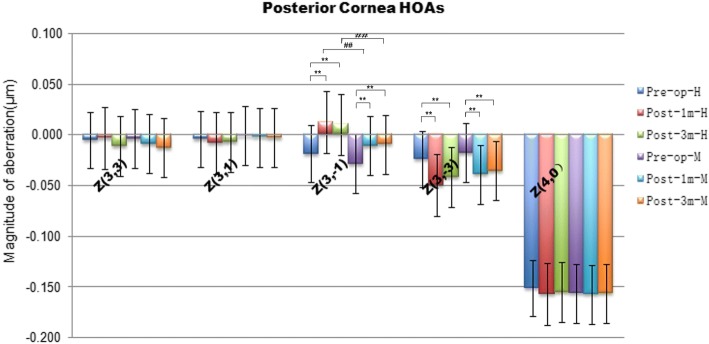
Higher-order aberrations (HOAs) of the posterior cornea surface before and after SMILE surgery in Group H and group M. *p, ^#^*p* < 0.0167 (the modified Bonferroni correction, 0.05/3 tests)

There was no significant difference in spherical aberrations (Z^4, 0^) of the posterior cornea between the two groups preoperatively or postoperatively (*p* > 0.0167). There was also no significant increased in spherical aberration in the two groups after smile surgery (*p* > 0.0167). There was no significant differences between-group in coma aberrations preoperatively (*p* > 0.0167). However, there were significant changes in vertical coma (z^3,-1^) and vertical trefoil (Z^3, − 3^) after SMILE surgery in the two groups (*p* < 0.167). In addition, there was significant difference in vertical coma between the two groups after surgery (*p* < 0.167) (Fig. 3).

### Comparison of surgically induced total corneal HOAs in group H and group M

**Fig. 4:**
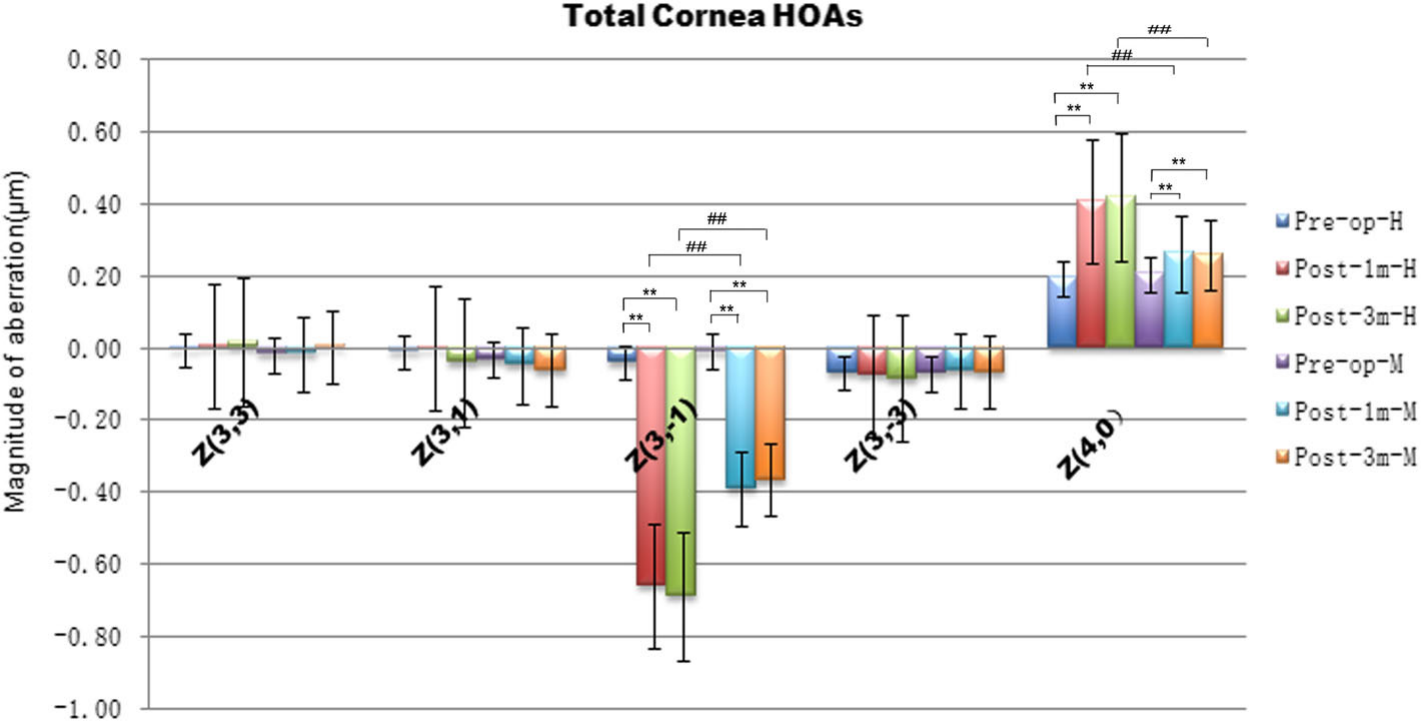
Higher-order aberrations (HOAs) of the total cornea before and after SMILE surgery in Group H and group M. *p, ^#^*p* < 0.0167 (the modified Bonferroni correction, 0.05/3 tests)

There were no significant differences in total corneal HOAs (coma and spherical aberrations) between the two groups preoperatively (*p* > 0.0167). In contrast, there were significant differences in vertical coma (Z^3,-1^) and spherical aberrations (Z^4, 0^) between the two groups 1 and 3 months postoperatively (*p* < 0.0167). More total corneal coma and spherical aberrations were induced in Group H than Group M postoperatively (*p* < 0.0167) (Fig. 4).

### Correlations between the preoperative SE and surgically induced corneal HOAs

Correlations between the preoperative SE and surgically induced corneal HOAs are shown in Table 3. The SE was significantly correlated with the induction of HOAs (third-order to eight-order, RMS HOAs) of the anterior surface and total cornea, especially vertical coma and spherical aberrations after SMILE surgery (*p* < 0.05) (Table 3).

**Table 3:** Correlations between SE and induced corneal HOAs after SMILE surgery

Cornea aberrations	*r*	*p*
RMS HOA (CF)	−0.631	0.000
RMS HOA (CB)	0.011	0.867
RMS HOA (cornea)	−0.648	0.000
Oblique trefoil Z^3,3^ (CF)	0.047	0.516
Horizontal coma Z^3.1^ (CF)	0.028	0.692
Vertical coma Z^3.-1^ (CF)	0.616	0.000
Vertical trefoil Z^3.*-3*^ (CF)	0.076	0.290
Spherical aberration Z^4,0^ (CF)	−0.582	0.000
Oblique trefoil Z^3, 3^ (CB)	−0.046	0.517
Horizontal coma Z^3, 1^ (CB)	0.026	0.718
Vertical coma Z^3, − 1^ (CB)	−0.323	0.000
Vertical trefoil Z^3, −3^ (CB)	−0.060	0.398
Spherical aberration Z^4, 0^ (CB)	0.037	0.601
Oblique trefoil Z ^3, 3^ (cornea)	0.027	0.702
Horizontal coma Z^3,1^ (cornea)	0.026	0.715
Vertical coma Z^3, −1^ (cornea)	0.613	0.000
Vertical trefoil Z^3,-3^ (cornea)	0.070	0.329
Spherical aberration Z^4, 0^ (cornea)	0.561	0.000

RMS-HOA-CF: third-order to eight-order aberrations of anterior corneal surface (cornea front, CF); RMS-HOA-cornea: third-order to eight-order aberrations of total corneal surface; RMS-HOA-CB: third-order to eight-order aberrations of posterior corneal surface (cornea back, CB)

## Discussion

This study is the first to compare corneal HOAs of the anterior surface, posterior surface, and total cornea in high and mild to moderate myopic eyes after SMILE surgery. The results indicated that HOAs of the anterior cornea surface and total cornea significantly increased after SMILE surgery in both groups. However, HOAs of the posterior corneal surface remained relatively stable. These results are consistent with those of other studies, which indicated that posterior corneal HOAs could complement corneal topography information, provide insight into the corneal biomechanical response, and provide valuable information that could be used to determine the cause of poor vision quality after corneal refractive surgery [2, 3].

This study revealed no significant differences in anterior cornea surface, posterior cornea surface and total corneal HOAs between high and mild to moderate myopic groups before SMILE surgery. However, significant between-group differences in anterior cornea surface HOAs and total corneal HOAs were detected postoperatively. In terms of SMILE-induced HOAs, there were significantly more HOAs in the high myopic group than mild to moderate group postoperatively, whereas HOAs of the posterior cornea remained almost unchanged postoperatively in both groups. The lack of significant changes in the posterior corneal surface is in agreement with that of previous studies, which examined the posterior corneal surface after PRK (Photo Refractive Keratectomy), FS-LASIK, and SMILE using Pentacam [3, 12, 13]. Wu et al. compared HOAs after SMILE, FS-LASIK, and FLEx (Femtosecond Lenticule Extraction) surgeries [3]. They reported a value of 0.19 ± 0.03 μm for third-order to eight-order aberrations of the posterior corneal surface in each surgical group postoperatively. They found that these HOAs not only remained stable postoperatively but were similar to values for HOAs obtained prior to surgery. In the present study, the number of third-order to eight-order aberrations of the posterior corneal surface in each group preoperatively and postoperatively was similar to that found by Wu [3]. Gyldenkerne et al. reported that the posterior corneal surface showed almost no change after FS-LASIK and SMILE procedures, which was consistent with our study [13]. Juhasz et al. analyzed changes in anterior and posterior corneal surface HOAs after PRK [12]. They found that PRK-induced HOAs increased significantly more than 76.78 μm ablation depths. And the HOAs on the anterior corneal surface increased total corneal aberrations. However, posterior corneal surface HOAs remained relatively stable after surgery. They also pointed out that aberrations of the anterior corneal surface were statistically significantly higher than that of the total cornea, indicating that the posterior corneal surface plays a compensatory role in the balance of corneal aberrations in myopic eyes.

This study also found that spherical aberrations induced by the SMILE procedure increased in the anterior corneal surface and total cornea. The induction of spherical aberrations was significantly greater in the high myopic group than mild to moderate group. However, spherical aberrations of the posterior corneal surface remained almost unchanged in both groups after surgery. SMILE-induced coma aberrations, especially vertical coma aberrations of the anterior surface, posterior surface, and total cornea changed in both groups, with a significantly greater increase in vertical coma aberrations in the high myopic group than mild to moderate group. These results were similar to study of Wu et al. [3] Their study indicated that FS-LASIK, FLEx, and SMILE surgeries induced spherical aberrations and coma of the anterior surface and total cornea. The SMILE surgery induced fewer spherical aberrations of the anterior cornea and total cornea than FLEx procedure, and posterior corneal spherical aberrations significantly increased after FS-LASIK surgery. Thus, the SMILE surgery seems to induce fewer posterior corneal coma aberrations as compared with the FLEx surgery. Another study that compared aberrations induced by FLEx and wavefront-guided LASIK procedures indicated that FLEx induced fewer spherical aberrations but the same amount of coma aberrations as compared with that observed using wavefront-guided LASIK1 [14]. Gertnere et al. found a reduced incidence of spherical aberrations in a FLEx group but a higher incidence of induced coma aberrations in an FS-LASIK group [15]. Lin et al. compared changes in aberrations induced by FS-LASIK and SMILE surgery and found a significantly lower incidence of spherical aberrations in the SMILE procedure [16]. The aforementioned studies used different LASIK ablation techniques and different microkeratomes for cutting the LASIK flap. The use of different LASIK ablation techniques and diverse methods of flap creation may result in substantial differences in HOAs [13]. The increased number of induced aberrations observed with the FS-LASIK procedure seems to be primarily associated with the ablation of corneal tissue rather than the creation of the flap, as the flap-dependent FLEx procedure does not seem to be different from that used in SMILE [13]. In the present study, there were significantly more surgically induced aberrations in the high myopia group than mild and moderate myopia group, and more central corneal tissue was removed in the high myopia group than mild to moderate myopia group. The correlation study also indicated changes in the induction of aberrations in the two groups were related to the preoperative SE, which was consistent with the findings of the study by Chen et al. [17].

Previous studies indicated that centeration and wound healing might influence the induction of coma aberrations [18, 19]. Li et al. demonstrated that horizontal decentration induced horizontal coma aberrations but that there appeared to be no association between the magnitude of vertical decentration and induction of vertical coma aberrations [20]. In our previous study, we reported that spherical aberrations and horizontal coma aberrations increased significantly after SMILE surgery and that the increase of spherical aberrations was higher in Group H than that in Group M [8]. This result was similar to findings presented by Liang et al. [21]. The difference in these results may be due to different methods used to evaluate corneal aberrations. In our previous study, we used a Hartmann–Shack WASCA aberrometer (Carl Zeiss Meditec AG, Jena, Germany). To optimize postoperative vision quality, more studies are needed to investigate the resources of the vertical coma and horizontal coma.

It is known that tear film problems that might influence the measurement of the aberrations of the anterior corneal surface, especially in dry eyes. Recently, Jung reported that total HOA RMS, coma and trefoil significantly increased at 10 s after blinking compared with those measured immediately after blinking in dry eye patients after LASEK (Laser Assisted Subepithelial Keratomilesusis) [22]. In the study of Elmohamady [23], they evaluated dry eye after LASIK, FS-LASIK, and SMILE. They found the mean ocular surface disease index (OSDI) scores were significantly elevated in all groups postoperatively but were significantly lower in the SMILE group 3 months postoperatively. The mean tear breakup time (TBUT) was significantly decreased in all groups postoperatively but was significantly higher in the SMILE group 6 months postoperatively. This result indicated the influence on dry eye was minimal after SMILE surgery. In our study, there were no significant differences in aberrations in the two groups after SMILE surgery 1 and 3 months postoperatively. This result may be due to the relatively small interference of SMILE on the tear film. Of course, long-term follow-up is still needed.

There were some limitations in this study. First, this study included 197 eyes, the two groups were not of equal size, and available data covered only 3 months. Longer term follow-up visits would have been desirable. Second, for bilaterally treated patients, there may be a correlation between the two eyes of one patient. This is a common mistake in ophthalmology research, for the overall variance of a sample of measurements combined from both eyes is likely to be an underestimate of the true variance resulting in an increased risk of a Type 1 error [24]. Future studies of the association of HOAs and corneal biomechanics with vision quality are needed to shed light on.

In conclusion, third-order to eight-order aberrations, particularly spherical aberrations and vertical coma aberrations of the anterior cornea and total cornea significantly increased after SMILE surgeries. In contrast, posterior corneal surface HOAs remained relatively unchanged. The induction of aberrations postoperatively was related to the preoperative SE. Further and larger studies, with longer-term follow-ups are needed.

## Abbreviations

CB: Posterior cornea surface/Cornea Back
CF: Anterior cornea surface/Cornea Front
FLEx: Femtosecond lenticule extraction
FS-LASIK: Femtosecond laser-assisted LASIK
HOAs: High-order aberrations
LASEK: Laser Assisted Subepithelial Keratomilesusis
LASIK: Laser-assisted in situ keratomileusis
OSDI: Ocular surface disease index
PRK: Photo Refractive Keratectomy
RMS: Root mean square
SE: Spherical equivalent
SMILE: Small incision lenticule extraction
TBUT: Tear breakup time
